# The impact of repetitive oocyte retrieval on the ovarian reserve: a retrospective cohort study

**DOI:** 10.1007/s00404-019-05098-9

**Published:** 2019-02-21

**Authors:** Julian Marschalek, Johannes Ott, Matthias Aitzetmueller, Daniel Mayrhofer, Andrea Weghofer, Kazem Nouri, Katharina Walch

**Affiliations:** Department of Obstetrics and Gynecology, Medical University of Vienna, Waehringer Guertel 18-20, 1090 Vienna, Austria

**Keywords:** Anti-Müllerian hormone (AMH), Oocyte retrieval, Follicular puncture, Ovarian reserve, IVF/ICSI

## Abstract

**Purpose:**

To investigate a possible influence of repetitive micro-traumata on the ovaries in the course of oocyte retrieval during IVF/ICSI treatment on serum anti-Müllerian hormone (AMH) levels.

**Methods:**

The study included retrospectively collected data from women who underwent three or more consecutive IVF/ICSI treatments between 2007 and 2017. The primary endpoint of the study was to evaluate changes in serum AMH levels on cycle days 1–3 during the course of repetitive IVF/ICSI treatments.

**Results:**

A total of 125 patients were included in this study. Median AMH levels before the first, second and third IVF/ICSI cycles were 3.8 ng/mL (IQR 1.8–7.1), 3.3 ng/mL (IQR 1.8–6.1) and 3.0 ng/mL (IQR 1.6–5.3), respectively (*p *= n.s.). In patients who underwent IVF/ICSI due to polycystic ovary syndrome (PCOS), we found a significant decrease in AMH serum levels between the first [AMH 9.7 ng/mL (IQR 7.4–14.4)] and the third [AMH 5.3 ng/mL (IQR 3.3–10.4)] IVF/ICSI cycles (*p* = 0.026). When performing a generalized linear model, we found PCOS to be an independent predictor for serum AMH decrease during the course of three oocyte retrievals (*p* < 0.001).

**Conclusions:**

When comparing the indications for IVF/ICSI, we observed a significant decrease in AMH serum levels after repetitive oocyte retrievals only in women with PCOS, while the decrease in AMH was not significant in patients with tubal factor, endometriosis, male factor and unexplained infertility. This finding leads us to hypothesize that repetitive micro-traumata on the ovarian cortex might diminish/normalize functional ovarian reserve in women with PCOS. Further prospective studies are highly warranted to allow firm conclusions.

## Introduction

The anti-Müllerian hormone (AMH) is probably the best marker of ovarian reserve at the moment and is reported to be highly sensitive to changes that accompany advancing age [1, 2] or iatrogenic ovarian damage [3, 4].

In women with clomiphene citrate-resistant polycystic ovary syndrome (PCOS) laparoscopic (LOD) or transvaginal ovarian drilling (TOD) serves as an effective surgical treatment option to lower AMH levels postoperatively [5]. The amount of energy used in LOD is discussed to be a crucial factor for the extent of the fall in serum AMH concentration. Whether this decrease reflects a real damage to ovarian reserve is still a matter of discussion, but some authors suggest that any surgical intervention, that leads to loss of ovarian follicles, might be associated with subsequent reduction in AMH [6].

The current pregnancy rates in in vitro fertilization (IVF) and intracytoplasmatic sperm injection (ICSI) make repetitive oocyte retrievals oftentimes necessary [8]. Moreover, and especially in women with a high response to controlled ovarian hyperstimulation (COH), it might be necessary to set several micro-traumata during oocyte retrieval—to gather every follicle and oocyte and subsequently reduce estrogen levels. Whether repetitive oocyte retrievals in women undergoing IVF/ICSI treatment lead to significant ovarian damage and reduction in AMH has—so far—not been studied.

To the best of our knowledge, this study is the first to investigate a possible influence of repetitive micro-traumata on the ovaries during IVF/ICSI treatment on serum AMH levels.

## Materials and methods

### Study population and study design

The study included retrospectively collected data from all the women who underwent three or more consecutive IVF/ICSI cycles between September 1, 2007, and September 1, 2017, at the Medical University of Vienna, Department of Obstetrics and Gynecology, Division of Gynecological Endocrinology and Reproductive Medicine. The primary endpoint of the study was to evaluate changes in serum AMH levels on cycle days 1–3 before COH during the course of repetitive IVF/ICSI treatments.

Inclusion criteria were: age over 18 years, three or more consecutive IVF/ICSI treatments, consecutive serum AMH values at cycle days 1–3 before COH. Women with missing data or incomplete primary or secondary outcome parameters were excluded from the analyses.

The diagnosis of polycystic ovary syndrome was based on the Rotterdam criteria [8].

### Sample analysis

AMH serum parameters were determined after blood sampling in the ISO-certified central laboratory of the Medical University of Vienna, Vienna, Austria, using commercially available radioimmunoassays (AMH; DSL Active MIS/AMH assay; Beckman Coulter Inc., Brea, USA). All AMH values before August 23rd, 2013 were corrected using the following formula *y* = 2.01 × *x* (R2 = 0.98). This had been due to a complement interference problem of the Beckman AMH ELISA before August 2013, when a dilution protocol had been implemented [9].

### Statistical analysis

Statistical analyses were performed with the SPSS software package, version 24.0 (SPSS, Chicago). Numerical data are as median (interquartile range, IQR), categorical data are presented numbers (frequencies). Differences between groups were tested using the Fisher’s exact test for nominal variables and the Welch’s test for numerical variables. A generalized linear model was used to test possible influencing factors on AMH. For this analysis, *β* values with their standard deviations, Wald’s test and likelihood ratio tests are provided. Differences were considered significant if *p* < 0.05.

The ethical review board of the Medical University of Vienna approved the study (EK 044/2010), which was performed in accordance with the Declaration of Helsinki and the guidelines of Good Scientific Practice, as supported by the Head of the Institute. As this study comprises retrospectively collected and analyzed data, the ethical review board approved the waiver of written informed consent.

## Results

A total of 125 patients fulfilled all the inclusion criteria and were included in this study. Patient characteristics are provided in Table 1. Median AMH levels before the first, second and third IVF/ICSI cycles were 3.8 ng/mL (IQR 1.8–7.1), 3.3 ng/mL (IQR 1.8–6.1) and 3.0 ng/mL (IQR 1.6–5.3), respectively. The dynamics in median AMH values between the IVF/ICSI cycles (delta AMH) did not reach statistical significance (*p *= n.s.). The interval between the first and the second, and the second and the third oocyte retrievals were 118.5 (73.75–216.50) days and 173.5 (117.75–258.75) days, respectively.

**Table 1:** Patient characteristics of 125 women undergoing three oocyte retrievals in the course of IVF/ICSI

	All women (*n* = 125)	PCOS^b^ (*n* = 16)	*p* value	Endometriosis^b^ (*n* = 17)	Tubal factor^b^ (*n* = 22)	Male factor^b^ (*n* = 91)
Female age (years)	33.2 (4.7)	30.9 (4.7)		33.8 (4.1)	33.7 (3.8)	33.2 (4.8)
Female BMI^a^ (kg/m^2^)	24.1 (5.6)	25.1 (6.1)		23.4 (3.9)	24.7 (6.1)	24.1 (5.6)
Nicotine abuse	36 (28.8)	2 (12.5)		3 (17.6)	4 (18.2)	29 (31.9)
Indication for IVF/ICSI^b^	–					
PCOS	16 (12.8)	–		1 (5.9)	1 (4.5)	8 (8.8)
Endometriosis	17 (13.6)	1 (6.3)		–	2 (9.1)	7 (7.7)
Tubal factor	22 (17.6)	1 (6.3)		2 (11.8)	–	5 (5.5)
Male factor	91 (72.8)	8 (50.0)		7 (41.2)	5 (22.7)	–
Unexplained infertility	2 (1.6)	–		–	–	–
Median AMH (ng/mL)						
Before IVF/ICSI cycle 1	3.76 (1.74–7.14)	9.71 (7.43–14.35)		3.14 (1.52–4.60)	2.49 (1.11–4.33)	3.84 (1.79–7.05)
Before IVF/ICSI cycle 2	3.27 (1.84–6.05)	6.70 (5.10–14.17)	0.026^d^	2.77 (1.09–5.32)	2.16 (1.27–5.13)	3.28 (2.06–6.07)
Before IVF/ICSI cycle 3	3.01 (1.62–5.26)	5.29 (3.33–10.39)		2.61 (1.33–5.72)	1.87 (0.99–3.53)	2.95 (1.74–5.29)
Median FSH (mIU/mL)^c^						
Before IVF/ICSI cycle 1	6.20 (4.4–8.1)	5.3 (3–6.5)		6.7 (4.3–9.1)	7.3 (5–8.8)	5.8 (4.4–8.2)
Before IVF/ICSI cycle 2	6.8 (5.3–8.1)	5.8 (4.5–6.6)		7.2 (5.5–9.8)	6.8 (6–8.4)	6.9 (5.3–8)
Before IVF/ICSI cycle 3	6.6 (4.8–8.1)	6.6. (3.3–7.8)		6.8 (3.7–8.1)	7.4 (6.1–8.7)	6.3 (4.5–8.3)
Median LH (mIU/mL)^c^						
Before IVF/ICSI cycle 1	4.6 (3.5–6.1)	6.1 (3.2–11.5)		3.9 (1.6–6.1)	4.9 (4.1–7)	4.5 (3.5–5.9)
Before IVF/ICSI cycle 2	5.1 (3.9–6.4)	6.3 (3.3–11.3)		5.4 (3.9–5.9)	4.2 (3.1–5.9)	5.2 (4–6.6)
Before IVF/ICSI cycle 3	5.2 (3.7–7.2)	7.4 (1.5–15.3)		5.6 (4–7.2)	5.8 (3.5–7.9)	5.2 (3.8–7.3)
Median Estradiol (pg/mL)^c^						
Before IVF/ICSI cycle 1	41 (31–62)	36 (27.8–58)		36 (14–70)	48.5 (34.3–76.3)	41 (32.3–61.3)
Before IVF/ICSI cycle 2	38 (27.5–54)	41 (34–138)		38.5 (30–62.5)	35 (27–48.3)	38.5 (28–52)
Before IVF/ICSI cycle 3	36 (29–57)	32 (29–57)		31 (10–60)	43.5 (31.5–66)	36 (29–54)
Stimulation protocol^e^	–					
Antagonist protocol	326 (86.9)	43 (89.6)		40 (78.4)	53 (80.3)	244 (89.4)
Long protocol	49 (13.1)	5 (10.4)		11 (21.6)	13 (19.7)	29 (10.6)
Retrieved oocytes^e^						
IVF/ICSI cycle 1	5 (2.5–9)	9 (7–16)		4 (3–8)	5 (2–10)	5 (2–9)
IVF/ICSI cycle 2	7 (5–10.5)	10.5 (6.25–19.25)	n.s.^f^	6 (4.25–13.25)	7 (4–9)	8 (5–10.75)
IVF/ICSI cycle 3	8 (5–15)	15 (5–17.5)		6 (4.5–12.5)	8 (5–15)	8 (5–14)

Data are shown for the whole study population as well as per main indication groups

Categorical data are presented as the frequency and percentage. Continuous variables are expressed as the median and interquartile range (IQR)

^a^BMI body mass index

^b^Multiple diagnoses possible

^c^Median FSH, LH, estradiol levels were measured in early follicular phase

^d^Decrease in AMH between IVF/ICSI cycles 1–3

^e^Data calculation per attempt (and not per patient)

^f^Difference in retrieved oocytes between IVF/ICSI cycles 1–2 and 1–3

**Fig. 1:**
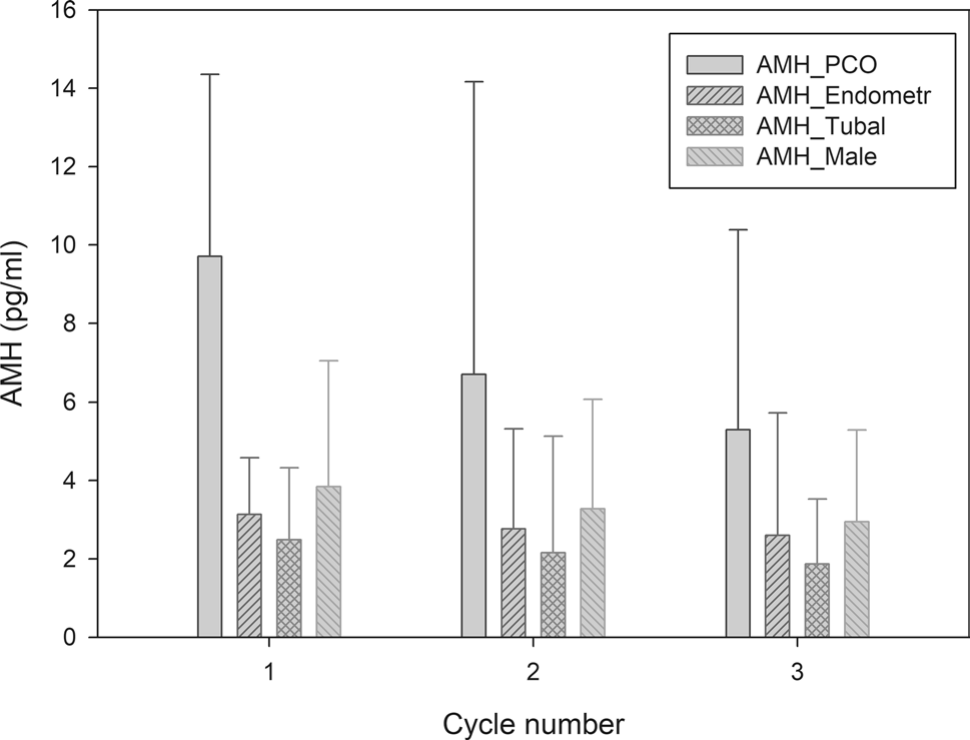
AMH (ng/mL) dynamics and indication for IVF

When comparing indications for IVF/ICSI, we found no significant difference with respect to AMH dynamics in patients with tubal factor, endometriosis, male factor or unexplained infertility (*p *= n.s.). In patients that underwent IVF/ICSI due to PCOS, we found a significant decrease in AMH serum levels between the first and the third cycles (*p = *0.026) (Table 1, Fig. 1).

In a next step, we performed a generalized linear model for the prediction of AMH dynamics (Table 2). We found PCOS to be an independent predictor for serum AMH decrease during the course of oocyte retrievals (*p < *0.001). We could not assess correlations between a decrease in AMH serum levels and tubal factor, endometriosis, male factor, age and BMI between the IVF/ICSI cycles (*p *= n.s.).

**Table 2:** Generalized linear model for the prediction of AMH dynamics (delta AMH before IVF/ICSI cycle 1—AMH before IVF/ICSI cycle 3)

Parameter	*B*	SE	Wald–Chi-square	*p* value
Tubal factor	0.913	9.324	0.997	0.318
Endometriosis	1.501	1.022	2.157	0.142
PCOS	− 3.530	1.004	12.350	< 0.001
Male factor	− 0.052	0.926	0.003	0.955
Age	− 0.044	0.649	0.457	0.499
Female BMI	− 0.013	0.060	0.045	0.831
Time interval (days)	− 0.001	0.001	0.418	0.518

*B* regression coefficient, *SE * standard error, *PCOS* polycystic ovary syndrome, *BMI* body mass index

## Discussion

In this study, we aimed to investigate a possible influence of repetitive oocyte retrievals during IVF/ICSI treatment on serum AMH levels. When comparing the indications for IVF/ICSI, we observed a significant decrease in AMH levels only in women with PCOS, while the decrease in AMH was not significant in patients with tubal factor, endometriosis, male factor and unexplained infertility.

AMH is described to be a useful parameter for the evaluation of iatrogenic ovarian damage: Chang and Iwase at first reported a decline of serum AMH levels after surgery for endometrioma [4, 10] and during the past 2 decades, a lot of other studies confirmed, that AMH levels decrease after invasive ovarian surgery [3, 11]. It is reported that the amount of decrease in AMH seems to be depending on the extent of ovarian damage during surgery [13–15]. Some authors suggest, that any surgical damage to the ovary might be associated with a subsequent loss of ovarian follicles followed by a decrease in AMH serum levels [6].

Whether repetitive oocyte retrievals have the potential to cause enough ovarian damage to be reflected in AMH serum levels has so far not been studied. Our results indicate a non-significant decrease in AMH serum levels during repetitive oocyte retrievals, especially in women with a normal or poor response to COH. By a median retrieval of six (four to ten) oocytes per follicular puncture, the harm of the ovaries might, therefore, not be reflected by AMH serum levels.

In contrast to normal or poor responders, it is oftentimes necessary to set more micro-trauma during oocyte retrieval in women with a high response due to PCOS. It, therefore, appears logically consistent, that these women theoretically acquire more ovarian damage during oocyte retrieval than others. Our results support this theory as AMH levels decreased significantly in the course of repetitive oocyte retrievals, with a median AMH reduction from baseline of 3.0 ng/mL after the first and of 4.4 ng/mL after the second ovarian puncture. Whether this decrease in AMH serum levels really reflects a reduction of ovarian reserve or just an intervention-induced normalization of increased serum values remains unclear, and the literature does not provide a clear hypothesis.

To draw on a theory postulating that the decline in serum AMH after ovarian drilling might be caused by thermal damage [16, 17], the decrease in AMH serum levels after oocyte retrieval might be explained with a loss of primary, pre-antral and antral follicles due to a repetitive puncture and consecutive damage of the ovarian cortex.

Rather than anything else, transvaginal or laparoscopic ovarian drilling might serve as a comparative operative procedure from a technical perspective, even though no thermal damage is applied during oocyte retrieval. Of note, there have been raising concerns over a possible harming effect of LOD on ovarian reserve since some authors report an exceeded reduction in AMH serum levels after LOD [11, 18, 19].

Generally, ovarian drilling proved successful as second-line treatment for ovulation induction in clomiphene citrate-resistant women with PCOS [20, 21]. In their meta-analysis, Amer et al. performed a pooled analysis of 442 studies and reported a statistically significant AMH serum level reduction of 2.13 ng/mL after LOD [6]. Theoretically, it might be possible that repetitive oocyte retrievals and ovarian drilling have similar effects on the ovarian reserve. In 2010, Agdi and colleagues compared the results of a subsequent IVF treatment in women with PCOS after (unsuccessful) in vitro maturation (IVM) and women with PCOS without prior IVM. They reported previous ovarian puncture for IVM to be associated with a higher number of mature oocytes and embryos, but not to be associated with a better pregnancy rate. However, they did not assess AMH measurements in these patients [22].

One has to mention that the AMH decline in women with PCOS may not derive from a mechanical destruction alone. Ovarian hyperstimulation results in recruitment and, subsequently, growth of small AMH-producing follicles. Consequently, this would decrease AMH production [23]. Notably, reduced AMH expression following FSH treatment has already been demonstrated in vitro [24].

The progressive and age-related decline in AMH serum levels is reported to be 5.6% per year [25]. In our study, the mean time interval between the first and the third IVF/ICSI cycles was 172.2 ± 155.3 days. Although we consider the effect of repetitive oocyte retrieval to be substantial, we believe that repetitive oocyte retrievals should not be considered as a treatment option for women with PCOS. In the absence of follow-up AMH levels, no clinical conclusions can be made: whether the effect of repetitive oocyte retrievals has a long-lasting impact on ovarian reserve needs to be further evaluated.

Of course, our results have to be interpreted within its major limitation, namely the retrospective design. Another limitation is caused by the small sample size of our cohort. Due to the limited number of patients in the subgroups, patients with PCO or PCOS could be heterogeneous on the one hand, and moderate AMH changes might not reach statistical significance on the other. Thus, we cannot rule out significant AMH dynamics in subgroups other than PCOS, especially endometriosis. Furthermore—and due to a poor analytical performance—the use of the Beckman AMH ELISA might be seen as a minor limitation: we corrected values that were measured before August 2013 using a specific formula [9].

We consider it as a crucial strength that, to the best of our knowledge, our study is the first to investigate a possible influence of repetitive oocyte retrievals during IVF/ICSI treatment on serum AMH levels and ovarian reserve.

## Conclusion

When comparing the indications for IVF/ICSI, we observed a significant decrease in AMH serum levels after repetitive oocyte retrievals only in women with PCOS, while the decrease in AMH was not significant in patients with tubal factor, endometriosis, male factor and unexplained infertility. This finding leads us to hypothesize that repetitive micro-traumata on the ovarian cortex might diminish/normalize ovarian reserve in women with PCOS. Further prospective studies are highly warranted to allow firm conclusions.
